# Platelet-Rich Plasma for Wound Healing in Diabetic Patients

**DOI:** 10.3390/medicina61091535

**Published:** 2025-08-27

**Authors:** Elean Zanzov, Vanya Anastasova, Karina Ivanova, Petar Kiskinov

**Affiliations:** Section of Plastic, Reconstructive and Aesthetic Surgery and Thermal Trauma, Department of Propaedeutics of Surgical Diseases, Medical University Plovdiv, UMHAT “St. George”, 4001 Plovdiv, Bulgaria; elean.zanzov@mu-plovdiv.bg (E.Z.); vanya.anastasova@mu-plovdiv.bg (V.A.); karina.ivanova@mu-plovdiv.bg (K.I.)

**Keywords:** diabetic foot ulcer, platelet-rich plasma, chronic wounds, wound healing, autologous treatment, debridement

## Abstract

*Background/Objectives:* Diabetic foot ulcers (DFUs) are a common and serious complication of diabetes, often leading to infection, amputation, and reduced quality of life. Platelet-rich plasma (PRP) therapy has emerged as a promising treatment due to its potential to accelerate wound healing through growth factors and cytokines. Despite growing interest, evidence on PRP’s efficacy and safety in DFU management remains variable. This article critically reviews recent studies to evaluate the effectiveness of PRP in promoting ulcer healing, while examining methodological rigor, ethical considerations, and research parameters to provide a comprehensive, evidence-based assessment for clinical application. *Materials and Methods:* This review explores the biological mechanisms underlying platelet-rich plasma (PRP) as an adjunctive therapy for DFUs, focusing on its regenerative capabilities. PRP is an autologous concentration of platelets containing growth factors and bioactive molecules that promote angiogenesis, cellular proliferation, and extracellular matrix remodeling. Various application methods—topical, injectable, gel-based, and PRP-enhanced dressings—are examined. The review also evaluates the efficacy of PRP as monotherapy and in combination with other interventions such as debridement and split-thickness skin grafting. *Results:* Clinical studies suggest that PRP, particularly when used alongside surgical debridement or skin grafting, significantly enhances healing outcomes in patients with non-healing DFUs. It provides a biologically favorable environment for tissue regeneration while reducing inflammation and potentially exhibiting antimicrobial properties. However, variability in PRP preparation techniques, application protocols, and patient selection criteria presents challenges to standardization and broader clinical adoption. *Conclusions:* While PRP therapy demonstrates significant potential in the management of diabetic foot ulcers, further randomized controlled trials with standardized methodologies are essential to establish optimal treatment protocols and confirm long-term benefits. PRP offers a minimally invasive, autologous, and biologically active treatment modality that may serve as a vital component in the multidisciplinary approach to DFU management.

## 1. Introduction

Diabetic foot ulcers (DFUs) are among the most serious complications of diabetes mellitus, affecting up to one in four patients during their lifetime. These chronic, non-healing wounds significantly increase the risk of infection, lower-limb amputation, prolonged hospitalization, and premature mortality. The global burden of DFUs is rising in parallel with diabetes prevalence, creating a pressing need for effective and accessible treatment strategies. Despite advances in conventional care—including glycemic control, infection management, and mechanical off-loading—many DFUs remain refractory, leading to high recurrence rates and reduced quality of life.

Platelet-rich plasma (PRP), an autologous concentrate of platelets and growth factors, has gained attention as a potential adjunct in DFU management. By promoting angiogenesis, modulating inflammation, and accelerating tissue repair, PRP offers a biologically plausible and minimally invasive therapeutic option.

The objective of this article is to critically evaluate current evidence on the efficacy, safety, and mechanisms of PRP in the treatment of DFUs, identify gaps in the literature, and outline directions for future research.

## 2. Methods

This article is narrative literature review synthesizing evidence on the use of platelet-rich plasma (PRP) in the treatment of diabetic foot ulcers (DFUs). Relevant studies were identified from peer-reviewed journals, including randomized controlled trials, observational studies, case series, and mechanistic research. Sources were selected based on their direct relevance to PRP application in DFUs, covering monotherapy, combination therapies, biological mechanisms, and safety profiles. Data extraction focused on study design, sample size, patient population, intervention protocols, outcomes measured, and key findings. Critical appraisal considered methodological strengths and limitations, including randomization, blinding, follow-up duration, and risk of bias. No statistical meta-analysis was performed; instead, results were synthesized qualitatively to provide an integrated assessment of current evidence and gaps in knowledge.

## 3. Materials

### 3.1. Pathogenesis

#### 3.1.1. Diabetic Foot Ulcer (DFU)

The emergence of diabetic foot ulcers is a complex phenomenon resulting from various pathogenic factors amid poorly controlled and chronic diabetes mellitus. The global prevalence of DFUs in diabetes patients stands at 6.4% [1]. Approximately 50–60% of these individuals will experience a diabetic foot infection, and 15% may require some form of foot amputation due to infections related to bone or ulcers [2]. Key contributors to DFUs include diabetic neuropathy, vascular insufficiency, structural deformities, and wound infections.

#### 3.1.2. Diabetic Neuropathy

Neuropathy associated with diabetes affects all three components of the central nervous system: sensory, motor, and autonomic. Sensory neuropathy can lead to chronic injuries due to a lack of protective sensations such as pain and discomfort. Long-term hyperglycemia results in the upregulation of aldose-reductase and sorbitol dehydrogenase, which increases the production of fructose and sorbitol. These substrates are highly osmotic and can cause significant stress and damage to nerve cells [3]. Prolonged elevated blood glucose levels can also lead to non-enzymatic reactions with proteins, amino acids, and DNA, resulting in advanced glycation end-products (AGEs) and high levels of reactive oxygen species (ROS). Furthermore, autonomic nerve damage can lead to skin dryness, fissuring, and cracking, creating entry points for pathogenic bacteria [4]. The incidence of superficial skin fissures is reported to be three times higher in the diabetic population compared to non-diabetics [5]. Research indicates that denervated skin shows a reduced count of leukocytes due to impaired cell chemotaxis mediated by neuropeptides, as evidenced in various studies involving rat models, where denervated skin flaps displayed lower levels of macrophages and T-lymphocytes, contributing to delayed wound closure [6]. Motor neuropathy is a primary cause of foot deformities, limited joint mobility, and abnormal pressure points on the foot, leading to skin damage and callus formation. Additional signs include the loss of Achilles reflex, muscle atrophy, and paralysis.

#### 3.1.3. Peripheral Vascular Disease (PVD)

PVD is 2.5 to 3 times more prevalent in individuals with diabetes than in the non-diabetic population. Hyperglycemia and related alterations in glucose metabolism, such as AGEs and ROS, cause endothelial damage, hyperlipidemia, increased platelet activity, viscosity, and the development of atherosclerosis [7]. PVD in diabetic patients mainly affects vessels below the knee, particularly the posterior and anterior tibial arteries, with the femoral and popliteal arteries being less frequently involved [8]. Ischemic ulcers can arise from arterial occlusion when tissue perfusion is insufficient to maintain skin integrity. Microvascular changes also develop, resulting in poor microcirculation. Microvascular disease causes thickening of capillary walls, hyalinization of arterioles, and reduced vessel elasticity, leading to inadequate delivery of oxygen, nutrients, and antioxidants while hindering the outflow of metabolic byproducts [9]. Impaired angiogenesis plays a critical role in DFU development, as numerous studies indicate decreased levels of angiogenic growth factors such as vascular endothelial growth factor (VEGF-20) and fibroblast growth factor (FGF-2), which adversely affect the proliferative phase of wound healing. Moreover, hyperglycemia-induced endothelial dysfunction results in diminished nitric oxide synthesis and accelerated degradation [10].

#### 3.1.4. Structural Foot Changes

Motor neuropathy leads to inadequate muscle function, resulting in various deformities, including “claw” toe and hammer-toe contractures. Glycosylation of collagen in connective tissue correlates with increased stiffness, leading to permanent joint fixation in non-anatomical positions. These anatomical changes in the foot create new pressure distributions on the plantar surface, contributing to callus formation and ulcerations, as well as thinning of the fat pads beneath the metatarsal heads, resulting in skin injury at pressure points [11].

#### 3.1.5. Wound Infection

Infections associated with DFUs are significant contributors to high morbidity and mortality rates among diabetic patients, causing frequent discomfort and emotional distress. The diabetic population is predisposed to a higher incidence of diabetic foot infections (DFIs), which are often a consequence rather than a cause of foot changes. Factors directly linked to the development of DFIs include diabetic neuropathy (sensory, motor, and autonomic), peripheral vascular disease, immune deficiencies, and structural foot changes [12]. Poorly perfused skin and the reduced leukocyte count in denervated skin compromise local immune resistance, increasing susceptibility to infections in patients with long-standing diabetes. The nature of infections can range from cellulitis to limb-threatening infections, often involving polymicrobial flora common in this patient group. The risk of amputation rises when DFIs involve resistant bacteria, frequently stemming from unnecessary antibiotic use.

### 3.2. Current Treatment Modalities for Diabetic Wounds

#### 3.2.1. Infection Prevention

Foot infections are common and can have severe consequences. More than half of all foot ulcers become infected, which is a major factor leading to lower extremity amputations. The complications associated with microbial flora range from superficial cellulitis to chronic osteomyelitis and gangrene, necessitating careful management. Antibiotic therapy is not required for wounds without confirmed soft tissue or bone infections. Mild to moderate infections may require empiric therapy targeting Gram-positive cocci, while severe infections caused by drug-resistant organisms necessitate broad-spectrum antimicrobials aimed at aggressive Gram-negative aerobes and obligate anaerobes [12].

#### 3.2.2. Debridement

Debridement encompasses both non-mechanical (autolytic, enzymatic) and mechanical methods (sharp/surgical, wet-to-dry, aqueous high-pressure lavage, ultrasound, and maggot debridement therapy). It aims to remove nonviable tissue to facilitate wound healing and prevent serious complications.

#### 3.2.3. Enzymatic Debridement

This approach utilizes exogenous enzyme products to digest nonviable tissue, rather than relying solely on endogenous wound enzymes.

#### 3.2.4. Autolytic Debridement

This technique maintains a moist wound environment to facilitate endogenous enzymes in self-digesting nonviable tissue. Dressings that support this process include alginates, hydrocolloids, foams, films, and honey. Moist saline gauze is frequently used as a standard debridement method in studies.

#### 3.2.5. Mechanical Debridement

This includes sharp surgical debridement, wet-to-dry techniques, high-pressure lavage/irrigation, ultrasound debridement, and biosurgery. Mechanical debridement is a widely used conventional method for treating diabetic foot ulcers, with every procedure performed as if it may be the last, ensuring complete removal of nonviable tissues [13].

The clinical significance of wound debridement and ulcers with necrotic tissue, regardless of the infection status, cannot be overstated and should not be underestimated. Debridement for most wounds is considered a standard in wound management.

#### 3.2.6. Off-Loading

Various structural foot abnormalities are associated with increased levels of plantar pressure. In individuals with diabetes, deformities such as claw-toe and Charcot neuroarthropathy can significantly disrupt foot architecture, raising local pressures. The combination of foot deformities, loss of protective sensation, and inadequate off-loading leads to tissue damage and ulceration. Once an ulcer forms, studies indicate that healing may be chronically delayed unless the area is off-loaded, even if perfusion is adequate. Off-loading measures include wearing extra-depth shoes, post-operative shoes, felted foam dressings, removable walkers, and total contact casts (TCC) [14].

#### 3.2.7. Glucose Control

Controlling blood glucose levels is critical in treating DFUs. Patients should be educated on strict dietary guidelines and pharmacological adherence. The first-line pharmacological treatment for type 2 diabetes is Metformin; second-line options include SGLT-2 inhibitors, DDP-4 inhibitors, and GLP-1 receptor agonists; third-line treatments may involve combinations of these agents along with sulfonylureas or insulin. Specific combinations such as DDP-4 inhibitors with GLP-1 should be avoided. Adjustments may be needed based on the patient’s current medication regimen and response to treatment [15,16].

#### 3.2.8. Platelet-Rich Plasma (PRP)

Platelet-rich plasma is an autologous concentrate of platelets suspended in a small plasma volume, which can facilitate the healing process [17]. PRP preparation involves extracting a patient’s blood, followed by centrifugation to separate the platelets from other components. For PRP to be classified as “platelet-rich,” the platelet concentration must exceed baseline whole blood levels by at least five times [18]. Although activation of PRP is not mandatory, it can be enhanced by adding thrombin and calcium chloride, which promotes the release of growth factors and bioactive proteins from the platelets’ alpha granules. Various protocols exist that yield different PRP formulations with diverse concentrations of platelets, leukocytes, and growth factors, but the optimal formulation for diabetic wound treatment remains under investigation [19].

### 3.3. Multiple Methods Are Utilized for Applying PRP to Chronic Wounds

#### 3.3.1. Topical Application

The most common method, where liquid PRP is directly applied to the wound bed. The application frequency differs, with certain studies indicating that PRP can be used as a gel either once or twice a week [20]. A specific medical device designed with a plasma-like composition can keep PRP active for up to seven days, enabling patients to utilize growth factors at home [21]. In one investigation, the implementation of PRP therapy combined with daily topical application of the PRP resulted in an approximate 30% decrease in wound size over a span of 12 days [22].

#### 3.3.2. Injection

According to Deshmukh et al. PRP may be injected directly into the wound bed or around its margins for promoting wound healing in diabetic foot ulcers. The study conducted these injections on a weekly basis over a span of four weeks, followed by a one-month observation period. Findings indicated that 40% of the chronic ulcers achieved complete healing, while the remaining 60% exhibited healing progress after eight weeks [23].

#### 3.3.3. PRP Gel

PRP can form a gel-like substance for direct application to the wound, offering a sustained release of growth factors, providing a conducive environment for healing. PRP gel is created when activated PRP forms a gel that covers the wound, providing a conducive environment for healing [24]. PRP gel can be activated using several methods and the choice of activation method can influence the physical form of the PRP and the release of growth factors [25]. One of the most common methods is the use of thrombin and calcium chloride. Thrombin directly activates platelets, and calcium ions replenish those bound by the anticoagulant [26]. Soluble type I collagen can also be used to activate PRP. Studies suggest that collagen activation results in less clot retraction and equal release of PDGF-AB and VEGF compared to other methods [27].

As an alternative to calcium chloride, calcium gluconate can be used to trigger platelet activation and gel formation. PRP gel can be applied to the wound twice a week or once a week [20]. In one instance, the daily usage of PRP gel topically led to an approximately 30% reduction in wound size over 12 days [22]. Topical application of homologous platelet concentrate gel in healing wounds shows beneficial results in wound size reduction and induces granulation tissue formation [28]. It can also accelerate hemostasis, repair damaged tissue, and promote regeneration [29]. The resulting fibrin scaffold is crucial for the recruitment of monocytes, fibroblasts, and progenitor cells, enhancing tissue repair [25].

#### 3.3.4. PRP-Enhanced Dressings

Incorporating PRP into different wound dressings provides a continuous supply of growth factors. PRP-enhanced wound dressings integrate the advantages of conventional wound care products with the regenerative capabilities of platelet-rich plasma while being structured to release PRP in a dual fashion, incorporating additional agents such as antibiotics to improve healing in infected wounds [30]. These dressings are designed to deliver growth factors and other beneficial elements of PRP directly to the wound area in a controlled and sustained manner [31]. According to Kolimi et al. polymeric film-based dressings can be augmented with PRP [32]. PRP-enhanced dressings exhibit excellent biocompatibility, facilitate tissue repair and regeneration, and can be formulated with antibacterial properties [33].

## 4. Results and Critical Appraisal of Evidence

### 4.1. PRP as Monotherapy

Evidence on platelet-rich plasma (PRP) as a stand-alone treatment for diabetic foot ulcers (DFUs) remains limited and heterogeneous. Case reports and small uncontrolled series [34,35] have described encouraging outcomes, with partial or complete healing in a subset of chronic DFUs. However, these studies often involve small sample sizes (<30 patients), lack randomization, and use varying definitions of “healing,” which complicates interpretation.

The variability in PRP preparation methods—including differences in platelet concentration, leukocyte content, and activation protocols [36] further limits comparability. While the minimal risk profile of PRP makes it attractive for patients unable or unwilling to undergo surgical interventions, the absence of large randomized controlled trials (RCTs) leaves its efficacy as monotherapy uncertain. Current evidence should be considered hypothesis-generating rather than practice-changing.

### 4.2. RP Combined with Debridement

Several studies indicate a potential synergistic benefit when PRP is combined with surgical debridement. The debridement is often performed before PRP application. This prepares the wound bed by removing barriers to healing and exposing healthy tissue [37]. This creates a synergistic effect—the debridement creates a clean wound environment, while PRP provides growth factors to stimulate cell proliferation, angiogenesis, and tissue regeneration [38]. Jaseem et al. [39] conducted a 2-year prospective study in 20 patients (aged 40–75) with chronic or non-healing ulcers, reporting improved healing rates compared with historical controls.

However, the lack of a randomized comparator group limits causal inference. Liao et al. [40] performed a randomized study of 60 patients with refractory wounds, finding significantly improved healing rates at weeks 1 and 3 (*p* < 0.05) in the PRP + debridement group compared to conventional skin grafting. This study strengthens the evidence base through randomization but still suffers from short follow-up duration and unclear blinding, raising the risk of observer bias. Overall, while data suggests additive benefits, high-quality RCTs with standardized debridement protocols and longer follow-up are required to confirm durability of healing and assess recurrence risk.

### 4.3. PRP with Split-Thickness Skin Grafts (STSG)

The combination of PRP with STSG has been investigated in small interventional series. In one uncontrolled study [41], 8 of 9 patients achieved successful graft take following PRP application and graft placement. Tyagi et al. [42] reported an 85% reduction in graft loss and a 79% reduction in hematoma formation compared with historical controls, suggesting improved graft survival. However, both studies lack randomized design and are vulnerable to selection bias. Additionally, most reports fail to control confounding variables such as vascular status, glycemic control, and infection burden—all of which are critical determinants of graft success in DFUs [43]. While promising, current evidence is insufficient to make strong recommendations, and multicenter RCTs are needed.

### 4.4. Mechanistic Evidence and Ancillary Outcomes

Several preclinical and early clinical studies [20,44,45,46,47,48] support the biological plausibility of PRP in DFU healing via growth factor release, angiogenesis stimulation, and modulation of inflammation. Nevertheless, antibacterial effects remain less well-characterized, with only preliminary in vitro and animal data suggesting possible benefits.

Notably, outcome measures across trials vary widely—some report wound area reduction, others report time to complete closure—preventing direct pooling of results. Few studies assess patient-centered outcomes such as pain reduction, quality of life, or recurrence rates, which are essential for determining real-world impact.

## 5. Discussion

As previously noted, diabetic foot ulcers often exhibit slow healing rates, high recurrence risks, and contribute to increased amputation rates, significantly affecting individuals’ disability, hospitalization, and mortality. PRP facilitates healing through various mechanisms: growth factor stimulation, promotion of new blood vessel formation, modulation of inflammatory responses, potential antimicrobial properties, and influence on extracellular matrix remodeling. PRP supplies essential growth factors such as TGF-β, PDGF, KGF, HGF, VEGF, FGF, EGF, and IGF—all vital for tissue repair. Autologous PRP balances inflammation, matrix metalloproteinases, and tissue inhibitors, preserves growth factors and ECM, and reduces bacterial growth via leukocytes and antimicrobial peptides. Fibrin in Au-PRP also provides scaffolding for cell support and aids in wound contraction [44,45].

According to Deng J et al. the local application of rich platelet plasma can significantly improve the healing of diabetic wounds. Furthermore, the study found that 87.5% showed a significant benefit of using PRP to treat chronic diabetic wounds [46]. The mechanisms underlying PRP’s healing enhancement are attributed to the release of growth factors and cytokines that stimulate cell proliferation, ECM deposition, and neovascularization [20,46]. Although promising, evidence regarding PRP’s antibacterial and anti-inflammatory properties is more limited relative to its effects on vascularization and re-epithelialization [20,47]. Further research is required to clarify the precise mechanisms by which PRP exerts its therapeutic effects and to optimize preparation and application protocols for maximum clinical efficacy [48].

Numerous studies have examined PRP’s efficacy in treating diabetic wounds, with many showing improved healing rates and reduced healing times. However, the overall evidence remains heterogeneous. Well-structured, randomized controlled trials are necessary to establish PRP’s efficacy definitively and optimize treatment protocols [49,50,51,52].

The decision for treatment of DFUs with platelet-rich plasma should be considered based on several criteria, that will include or exclude a particular patient, some of with are absence of bacterial infection and necrotic tissue at the wound side, management of blood glucose levels, mechanical off-loading of the foot [53]. When these criteria are met, the patient is suitable for PRP treatment.

Bacterial infections in chronic diabetic wounds are common problem which should be managed properly before starting treatment with PRP, so the wound side will be suitable for the processes of angiogenesis, tissue proliferation and differentiation, chemotaxis of immune and structural cells, extracellular matrix formation [54]. There have been some reports for the possible presence of bacterial biofilm colonies surrounded by a protective coat of polysaccharides in chronic diabetic wounds that eventually disrupt the healing process if not eliminated radically [55]. The adequate treatment of the infection can be obtained by excision a tissue for cultural examination and choosing a suitable antibiotic treatment for the bacterial agent. Surgical debridement is another technique for radical elimination of infection, by removing the necrotic tissues, which are perfect environment for bacterial growth. Another part of making a DFU suitable for PRP treatment is the daily sterile moist dressing of the wound with exudate control [56].

Mechanical off-loading is strongly recommended because of the extensive changes in the foot’s anatomical structure, which causes areas with higher pressure on the plantar surface of the foot and micro traumatic events which lead to callus and ulcer formation. This offloading can be achieved by using total contact casts, removable cast walkers or Charcot restrain orthotic walkers which can improve the healing process [57].

A very important component is the strict blood glucose levels control, which will reduce the overall oxidative stress by lowering the levels of advanced glycation end products, which are observed in long duration uncontrolled diabetes mellitus and typically lead to worse wound reparation processes [58].

This systematic review highlights the potential of platelet-rich plasma (PRP) therapy in promoting healing of diabetic foot ulcers (DFUs). The findings indicate that PRP may accelerate wound closure, reduce infection rates, and improve tissue regeneration compared to conventional treatments.

Consistent with previous studies, our analysis supports the efficacy of PRP in enhancing angiogenesis and collagen deposition, which are critical for DFU healing. Some studies reported variable outcomes, likely due to differences in PRP preparation methods, treatment frequency, and patient comorbidities. This variability underscores the need for standardized protocols to optimize therapeutic outcomes.

Future research should focus on large-scale, randomized controlled trials with standardized PRP preparation and administration protocols. Investigating long-term outcomes and cost-effectiveness will also be critical for translating PRP therapy into routine clinical practice. While its use as a monotherapy is still under evaluation, evidence suggests that PRP can significantly enhance healing when combined with debridement [20] and split-thickness skin grafts [41]. Further research with standardized protocols is needed to fully elucidate the optimal applications and long-term benefits of PRP in wound management [59]. The clinical implications of these findings are significant. PRP therapy offers a promising adjunct to conventional wound care, potentially reducing the risk of amputation and improving quality of life for patients with DFUs. However, the heterogeneity of included studies, small sample sizes, and variations in follow-up duration limit the generalizability of the results.

## 6. Conclusions

Platelet-rich plasma (PRP) therapy shows promising results in the management of diabetic foot ulcers, demonstrating potential to accelerate wound healing, enhance tissue regeneration, and reduce complications.

Current literature supports PRP as a potential adjunct rather than a proven standard for DFU management.

Strengths: biological plausibility, favorable safety profile, preliminary clinical benefits in combination therapies.

Weaknesses: small sample sizes, lack of standardized PRP protocols, heterogeneous outcome definitions, and short follow-up periods.

Given these limitations, the findings should be interpreted with caution, and clinicians should consider PRP use within the framework of multidisciplinary DFU care rather than as a standalone definitive therapy.

While current evidence suggests clinical benefits, variations in study design and treatment protocols highlight the need for standardized approaches. Further high-quality research is essential to establish definitive guidelines and optimize the use of PRP in routine clinical practice, ultimately improving outcomes for patients with diabetic foot ulcers.

### Ethical and Research Parameters

The studies included in this review demonstrated varying levels of adherence to ethical and research standards. Most reported obtaining approval from Institutional Review Boards (IRBs) or Ethics Committees, in alignment with national regulations and international guidelines such as the Declaration of Helsinki. Participants provided informed consent, with additional parental or guardian consent obtained for studies involving minors. Measures were taken to ensure confidentiality and anonymity, including de-identification of personal data and secure storage of sensitive information.

## Data Availability

No new data were created or analyzed in this study. Data sharing is not applicable to this article.

## Abbreviations

PRP	Platelet-rich plasma
DFU	Diabetic foot ulcer
